# Toward automatic prediction of *EGFR* mutation status in pulmonary adenocarcinoma with 3D deep learning

**DOI:** 10.1002/cam4.2233

**Published:** 2019-05-10

**Authors:** Wei Zhao, Jiancheng Yang, Bingbing Ni, Dexi Bi, Yingli Sun, Mengdi Xu, Xiaoxia Zhu, Cheng Li, Liang Jin, Pan Gao, Peijun Wang, Yanqing Hua, Ming Li

**Affiliations:** ^1^ Department of Radiology Huadong Hospital Affiliated to Fudan University Shanghai China; ^2^ Diagnosis and Treatment Center of Small Lung Nodules Huadong Hospital Shanghai China; ^3^ Department of Electronic Engineering Shanghai Jiao Tong University Shanghai China; ^4^ SJTU‐UCLA Joint Center for Machine Perception and Inference Shanghai Jiao Tong University Shanghai China; ^5^ Diannei Technology Shanghai China; ^6^ Department of Pathology Shanghai Tenth People's Hospital, Tongji University School of Medicine Shanghai China; ^7^ Department of Radiology, School of Medicine Tongji Hospital, Tongji University Shanghai China; ^8^ Institute of Functional and Molecular Medical Imaging Fudan University Shanghai China

**Keywords:** convolutional neural networks, deep learning, *EGFR*, *mixup* training technique, radiomics

## Abstract

To develop a deep learning system based on 3D convolutional neural networks (CNNs), and to automatically predict EGFR‐mutant pulmonary adenocarcinoma in CT images. A dataset of 579 nodules with EGFR mutation status labels of mutant (Mut) or wild‐type (WT) was retrospectively analyzed. A deep learning system, namely 3D DenseNets, was developed to process 3D patches of nodules from CT data, and learn strong representations with supervised end‐to‐end training. The 3D DenseNets were trained with a training subset of 348 nodules and tuned with a development subset of 116 nodules. A strong data augmentation technique, *mixup*, was used for better generalization. We evaluated our model on a holdout subset of 115 nodules. An independent public dataset of 37 nodules from the cancer imaging archive (TCIA) was also used to test the generalization of our method. Conventional radiomics analysis was also performed for comparison. Our method achieved promising performance on predicting EGFR mutation status, with AUCs of 75.8% and 75.0% for our holdout test set and public test set, respectively. Moreover, strong relations were found between deep learning feature and conventional radiomics, while deep learning worked through an enhanced radiomics manner, that is, deep learned radiomics (DLR), in terms of robustness, compactness and expressiveness. The proposed deep learning system predicts EGFR‐mutant of lung adenocarcinomas in CT images noninvasively and automatically, indicating its potential to help clinical decision‐making by identifying eligible patients of pulmonary adenocarcinoma for EGFR‐targeted therapy.

## 1 INTRODUCTION

Lung cancer is one of the leading causes of cancer‐related death.^1^ Non‐small cell lung cancer (NSCLC) accounts for more than 80% lung cancer cases, where adenocarcinoma is the most common histological subtype.^2^ With the advancements of genomics, several genomic alternations, such as oncogenic *ALK* rearrangements,^3^ *ROS1* rearrangement,^4^ *KRAS* mutations,^5^ and sensitizing *EGFR* mutations,^6^ have been identified as predictive and prognostic markers for NSCLC. Targeted therapy with these molecular markers has become an essential part of precision medicine for lung cancer.

Small molecule tyrosine kinase inhibitors (TKIs) that target specific *EGFR* mutations have resulted in improved progression‐free survival (PFS) and higher objective radiographic response rate in patients with *EGFR* mutations than standard chemotherapy.^7^, ^8^, ^9^ Moreover, approximately 80% patients with *EGFR*‐mutant lung cancer respond to EGFR TKIs therapy (at initial treatment).^10^ However, the administration of EGFR TKIs (for example, gefitinib) on the patients without *EGFR* mutations, not just showed no effect but even resulted in worse PFS and unnecessary costs compared to platinum‐based chemotherapy,^11^ which highlights the importance of identifying eligible patients for the first‐line TKI therapy.

Mutation profiling after biopsies or surgical resections has become a standard and informative medical procedure. However, the high cost and invasiveness of the approaches and repeating tumor sampling strongly limit the applicability of molecular testing. Besides, the poor DNA quality, intratumoral heterogeneity and long turnaround time raise much concern on the balance of costs and benefits.^12^, ^13^ Notwithstanding re‐biopsy is feasible in clinical practice, it is an invasive operation and faces the same challenges voiced by previous studies.^14^, ^15^ These defects enormously limit the practicability of precision medicine at scale.

Alternatively, noninvasive markers are promising for predicting *EGFR* mutation.^16^, ^17^ Cancers with different genotypes drive specific biological processes involved in the development and progression of tumors, thus ultimately leading to different phenotypes. In other words, it is potentially feasible to predict the genotypes by identifying specific phenotypes. There have been studies investigating the phenotype‐genotype associations to identify associated genomic changes,^18^, ^19^ based on tumor morphology on computed tomography (CT)^20^, ^21^ and magnetic resonance imaging (MRI).^22^ Radiomics,^20^ that encodes tumor phenotypes with innumerable quantitative features using predefined image analysis algorithms. In particular, previous studies have demonstrated that certain radiomic features are associated with *EGFR* mutations status, suggesting that those identified features may be driven by somatic mutations.^16^, ^23^ Although these studies have achieved impressive performances, especially when combined with clinical information,^17^, ^23^, ^24^ conventional radiomics‐based methods are born with three main challenges. Firstly, conventional radiomics methods require strict procedures, including detection, segmentation, feature extraction, selection etc,^25^ which is tedious and time‐consuming. Secondly, radiomic features are susceptible to the manual segmentation as well as CT scanning parameters, thus interobserver reproducibility analysis is necessary. Finally, hand‐craft radiomic features are expressive but may be not enough for high‐level tasks.

On the other hand, deep neural networks, or *Deep Learning*, have achieved remarkable success in several important problems of artificial intelligence (AI), for example, natural image classification^26^ and human language translation.^27^ Deep convolutional neural networks (CNNs), a family of neural networks, have shown incredible effectiveness in several tasks of natural image computer vision and medical image computing.^28^, ^29^ As powerful algorithms of representation learning, CNNs largely reduce the necessities of hand‐craft feature engineering. Our previous study has proven the effectiveness and efficacy of deep learning in predicting the invasiveness of lung adenocarcinomas from CT images.^30^ In this regard, we addressed the problem of CT‐based *EGFR* mutation prediction by deep neural networks, to make our system automatic, robust and accurate.

In this study, we aimed to develop a deep learning system to predict the *EGFR* mutation status of lung adenocarcinoma based on CT images by integrating recent advances in deep supervised learning, such as dense connections^31^ and *mixup* training,^32^ to significantly reduce the empirical risks of overfitting. Our method is a labor‐saving strategy without the requirement of precise nodule segmentation, and also expected to obtain more stable performance due to the enhanced nature of the employed learning algorithms.

## 2 MATERIALS AND METHODS

**Figure 1:**
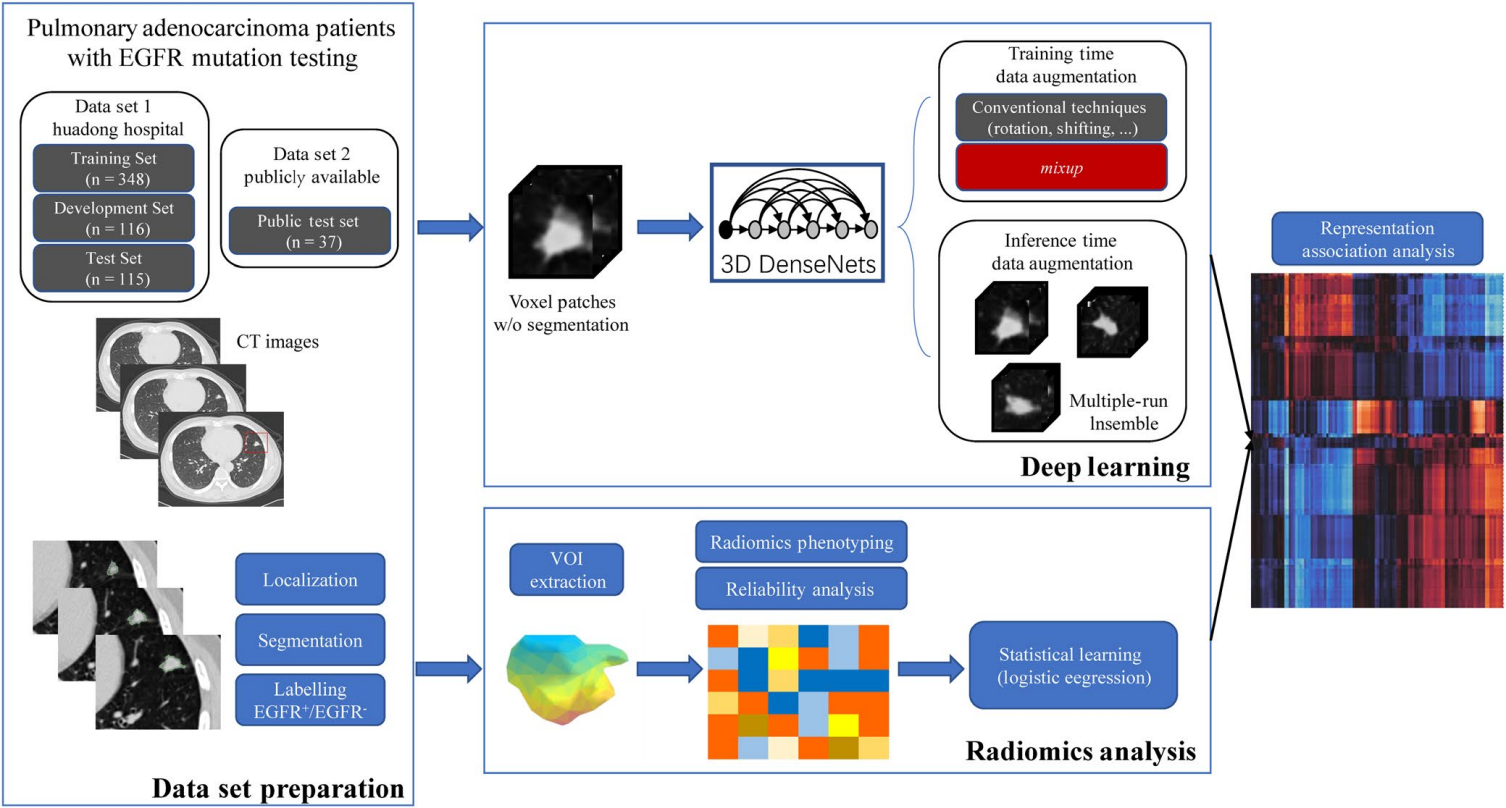
Overall pipeline for this study. A local CT dataset (HdH Dataset) and a public dataset selected from TCIA database (TCIA Dataset) of lung adenocarcinoma patients with *EGFR* mutation testing were used. Nodules were manually localized, segmented, and labelled as EGFR mutant (Mut)/wild‐type (WT). For deep learning, 3D DenseNets were trained using the training subset. A strong data augmentation technique, *mixup*, was used for better regularization. Expressive representations, that is, deep learned radiomics (DLR), for the nodules were end‐to‐end learned during the training procedure. Meanwhile, conventional radiomics analysis following the common practice was carried out for performance comparison, and association study between the 3D deep leaning and conventional radiomics was performed by calculating the pairwise correlation coefficients

We developed a novel analytic framework based on deep learning of the CT imaging phenotypes of lung adenocarcinoma to predict *EGFR* mutation status (see Figure 1). This retrospective study was approved by the Institutional Review Board of our institution (NO.20170103), which waived the requirement for patients' informed consent referring to the CIOMS guideline.

### 2.1 Data collection

This study used local data collected in Huadong Hospital (HdH Dataset) and publicly available data selected from TCIA (TCIA Dataset).^33^, ^34^ In HdH Dataset, the inclusion criteria were (a) patient receiving thin‐slice chest CT (0.75‐1.5 mm) scan prior to biopsies or surgical treatment, (b) with pathology reports for the diagnosis of pulmonary adenocarcinoma, and (c) with detailed *EGFR* mutation testing reports. Only one malignant nodule was studied for each patient due to the availability of *EGFR* testing report. Among the 579 lung adenocarcinoma patients, 308 were *EGFR‐*mutant (Mut) and 271 were *EGFR* wild‐type (WT). The detailed characteristics of patients and lesions in HdH Dataset are presented in Table 1. CT acquisition parameters are described in Appendix S1. In the TCIA Dataset, 37 nodules were selected to validate the stability and generalization of the analytic framework. The inclusion criteria were (a) CT with slice thickness ≤1.5 mm (to avoid data inconsistency), (b) with *EGFR* mutations testing reports, (c) with pathology reports for the diagnosis of lung adenocarcinoma, (d) and lesions that could be certainly identified as the resected or biopsied lesions. The detailed information of the TCIA Dataset is described in Table S1 **.**

**Table 1:** Characteristics of patients and lesions in HdH dataset

Characteristics	Number	Percentage
Gender		
Male	245	42.3
Female	334	57.6
Mean age (range) (y)		
Male	61.8 ± 11.6 (29‐85)	‐
Female	58.4 ± 11.9 (22‐85)	‐
Total	59.8 ± 11.9 (22‐85)	‐
Mean size (range) (cm)	1.8 (0.3‐8.6)	‐
Location		
Right lobe	342	59.1
Left lobe	237	40.9
Pathology		
Adenocarcinoma in situ	31	5.4
Minimally invasive adenocarcinoma	157	27.1
Invasive adenocarcinoma	391	67.5
TMN classification (eighth edition)		
0	31	5.4
I A‐B	356	61.5
II A‐B	7	1.2
III A‐C	10	1.7
IV A‐B	175	30.2
*EGFR* Mut	308	53.2

### 2.2 *EGFR* mutation profiling

Drug target‐associated mutations on *EGFR* exons 18, 19, 20, and 21 were examined using a PCR‐based amplification‐refractory mutation system (ARMS) with Human *EGFR* Gene Mutations Fluorescence Polymerase Chain Reaction Diagnostic Kit (ADx‐EG01, AmoyDx). Wild‐type *EGFR* in this study referred to no mutation detected among those loci.

### 2.3 Data annotation and pretreatment

Volumes of interest (VOIs) of the enrolled nodules in both datasets were manually delineated at voxel level by a radiologist with 4‐year experience in chest CT interpretation using medical image processing and navigation software 3D Slicer (version 4.8.0, Brigham and Women's Hospital), and subsequently confirmed (modified or re‐delineated) by another radiologist with 12‐year experience in chest CT interpretation. DICOM‐format images were imported into the software, subsequently the images with VOI information were exported into NII format for further analysis. Each segmented nodule was assigned a specific *EGFR* mutation label (Mut or WT) according to the corresponding *EGFR* mutation testing report.

Deep learning is known to require heavy tuning. Thus, we randomized the HdH Dataset into training, development, and test subsets, containing 60%, 20%, and 20% (only once) of each *EGFR* categories (Mut or WT), respectively. The training set was used for training the neural networks. The development set was used for tuning the hyperparameters, such as determining early stopping, discovering the most suitable neural architectures, and choosing the best model snapshots. Once we chose the best model using the development set, the holdout test set was used for fairly evaluating the performance of our method. To further validate the stability and generalization of our method, we evaluate our model on the independent TCIA Dataset, without any fine tuning.

### 2.4 *EGFR* mutation status prediction with conventional radiomics

We investigated the performance of the conventional radiomics method using the two datasets. Following the common practice of radiomics method,^23^ the radiomic features were extracted with numbers of image analysis algorithms. Specifically, 475 radiomics features, including 50 histogram features, 325 co‐occurrence matrix features, and 100 run lengths matrix features, were automatically extracted using MATLAB 2016b for all delineated nodules in the two datasets. The detailed radiomics extraction methodology is described in Appendix S1.

Radiomic features require refined manual segmentation of nodules. Even so, many radiomic features are not stable due to different segmentation manners. To ensure the reproducibility of radiomics, 50 nodules in the HdH Dataset were randomly selected for independent segmentation by two radiologists. The 475 radiomics features of the 50 nodules were evaluated with interclass correlation coefficient (ICC) analysis using the “irr” package in R software (version 3.4.3). Features with an ICC > 0.8 were considered reliable. Finally, a total of 401 features were selected.

Logistic regression was used to model the relation between the radiomic features and *EGFR* status with the widely used scikit‐learn library in Python.^17^, ^35^ Due to the simple hyperparameter setting of logistic regression, the training and development sets were merged into a “train‐dev” set. Ten‐fold cross validation search was performed on the train‐dev set with 1000 randomly sampled values of regularization term *C*. The trained model was used for scoring the HdH test Dataset and TCIA Dataset. We also tried heavy search for models and hypermeters with an automated machine learning tool (AutoML),^36^ built upon widely used scikit‐learn Python package^37^; however it did not yield better performance. Note that the feature selection procedure to reduce the redundancy between the features had been included in the AutoML search procedure. Although there may exist a better model theoretically, the heavy search process implied conventional radiomic features are not strong enough representations.

### 2.5 *EGFR* status prediction with 3D DenseNets

To take the advantage of deep representation learning, we designed a deep learning‐based framework for analyzing the *EGFR* mutation status of nodules. Taking into account the characteristics of the used data, we have adopted the following principle to design the models:

- *3D*: CT images were presented in 3D, and the 3D views provided critical visual information from a practical point of radiology;
- *Parameter‐efficient*: the model should be compact and easy to train with limited data;
- *Data‐efficient*: our training method should lead to less overfitting;
- *Automatic*: at inference stage, our model should be easy to use, given approximate locations of nodules, rather than the manual segmentation.

Following these principles, we adopted *3D DenseNets*, which were derived from the powerful deep convolutional neural networks 2D DenseNets.^31^ DenseNets have achieved great success in 2D natural images and medical images,^38^ which elegantly reuse the features from lower layers, thus exploring high‐level representations in an efficient way. Our 3D DenseNets used same notations but 3D convolutions, with a growth rate *k* = 16, a compression rate *θ* = 2, and a bottleneck *B* = 4. Batch Normalization^39^ and Leaky ReLU^40^ (*α* = 0.1) were used together as activation functions. The resulting neural networks contained only 0.55 M parameters, making the training compatible with limited data. We implemented the neural networks with Keras 2.1.5^41^ and TensorFlow 1.4.0.^42^ The detailed structure of the 3D DenseNets is depicted in Table 2.

**Table 2:** 3D DenseNet architectures for *EGFR* mutation classification

Layer	Tensor size	Building blocks
Input	48 × 48 × 48 × 1	
Convolution	48 × 48 × 48 × 32	3 × 3 × 3 conv
Pooling	24 × 24 × 24 × 32	2 × 2 × 2 average pool
Dense Block (1)	24 × 24 × 24 × 80	$\left[\begin{array}{c} \text{bn}-\text{leaky}\;\text{relu}-1\times 1\times 1\;\text{conv}\\ \text{bn}-\text{leaky}\;\text{relu}-3\times 3\times 3\;\text{conv} \end{array}\right]\times 3$
Compression and Pooling (1)	12 × 12 × 12 × 40	$\left[\begin{array}{c} \text{bn}-\text{leaky}\;\text{relu}-1\times 1\times 1\;\text{conv}\\ 2\times 2\times 2\;\text{average}\;\text{pool} \end{array}\right]$
Dense Block (2)	12 × 12 × 12 × 136	$\left[\begin{array}{c} \text{bn}-\text{leaky}\;\text{relu}-1\times 1\times 1\;\text{conv}\\ \text{bn}-\text{leaky}\;\text{relu}-3\times 3\times 3\;\text{conv} \end{array}\right]\times 6$
Compression and Pooling (2)	6 × 6 × 6 × 68	$\left[\begin{array}{c} \text{bn}-\text{leaky}\;\text{relu}-1\times 1\times 1\;\text{conv}\\ 2\times 2\times 2\;\text{average}\;\text{pool} \end{array}\right]$
Dense Block (3)	6 × 6 × 6 × 132	$\left[\begin{array}{c} \text{bn}-\text{leaky}\;\text{relu}-1\times 1\times 1\;\text{conv}\\ \text{bn}-\text{leaky}\;\text{relu}-3\times 3\times 3\;\text{conv} \end{array}\right]\times 4$
Compression and Pooling (3)	3 × 3 × 3 × 66	$\left[\begin{array}{c} \;\text{bn}-\text{leaky}\;\text{relu}-1\times 1\times 1\;\text{conv}\\ 2\times 2\times 2\;\text{average}\;\text{pool} \end{array}\right]$
Dense Block (4)	3 × 3 × 3 × 114	$\left[\begin{array}{c} \text{bn}-\text{leaky}\;\text{relu}-1\times 1\times 1\;\text{conv}\\ \text{bn}-\text{leaky}\;\text{relu}-3\times 3\times 3\;\text{conv} \end{array}\right]\times 3$
Global Pooling (*DLR*)	114	3 × 3 × 3 average pool
Output	1	sigmoid

The inputs of the proposed 3D DenseNets were cubic patches of 48 × 48 × 48 mm, generated by (pre‐processed) chest CT scans and the coordinates c=[z,y,x] of the approximate mass centers of nodule. In practice, the coordinates can be marked by radiologists manually, or by automatic nodule detection systems.^43^ Our method did not require the manually refined segmentation, which were usually done by the users (radiologists) manually. The preprocessing followed a “standard” procedure: the input patches were converted into Hounsfield units, followed by resizing the volumetric data into spacing of 1 × 1 × 1 mm by trilinear interpolation, clipping the voxel intensity into $I_{\text{HU}}\in \left[-1024,400\right]$, quantifying the density into grayscale, and transforming the values to $I\in \left[-1,1\right)$ by the mapping $I=\left[(\left(I_{\text{HU}}+1024\right)/\left(400+1024\right))\times 255\right]/128-1$. We further applied multiple data augmentation techniques to represent nodules in different views:

- Moving the centers by small amounts in [−*m*, *m*] pixels in the three axes
- Reordering the axes and rotation by 90° increments
- Left‐right flipping.

### 2.6 Training for the deep learning models

During training, we applied the above data augmentation techniques with *m* = 8. These conventional techniques could effectively increase the training data size, yet not enough for a good training convergence. We further used a novel data augmentation technique: *mixup* 32 to stabilize the training. *mixup* showed remarkable improvements in *state‐of‐the‐art* natural image classification neural networks. To our knowledge, this is the first comprehensive study introducing *mixup* into medical image computing.

The *mixup* produces extra informative training samples by the following easy‐to‐implement data augmentation routine. Given $\lambda \;\text{Beta}\left(\alpha ,\alpha \right)\in \left[0,1\right]$, *α* is a predefined hyperparameter, and (*x_i_*, *y_i_*) and (*x_j_*, *y_j_*) are two input‐label pairs from the training distribution, then extra training samples are obtained by the following equations:

$$\overset{¯}{x}=\lambda x_{i}+\left(1-\lambda \right)x_{j}$$

$$\overset{¯}{y}=\lambda y_{i}+\left(1-\lambda \right)y_{j}$$

*α* defines the strength of *mixup* by controlling the beta distribution; when *α* = 0, *mixup* ceases to be effective. In our experiments, we set *α* = 0.9 for modest usage of *mixup*. As shown later in the Results section, *mixup* was critical for enabling the training of deep neural networks with limited data on this task.

We trained the 3D DenseNets with binary cross‐entropy loss:

$$\ell \left(y,\hat{y}\right)=\frac{1}{n}\sum y\log\hat{y}+\left(1-y\right)\log\left(1-\hat{y}\right),$$

where *y* is the ground truth of the input, and $\hat{y}$ is the prediction given by the models. “Kaiming uniform” method^44^ was used for initializing the neural networks. During training, we sampled 20 positive and 20 negative samples (with a batch size of 40) for balanced optimization. A Nesterov‐momentum SGD^45^ with momentum = 0.9 was used as neural optimizer. The initial learning rate was 3 × 10^−2^, and decayed by a factor of 1/3 at the end of epoch = 30, 60, 100, and 150. No weight decay nor dropout^46^ was used. All hyperparameters were manually tuned on the HdH development set. A model snapshot was saved at the end of each epoch. Finally, the best model of a single run was selected by the following heuristics:

- initially, select five candidates with the highest AUCs on the development set;
- then, choose one of the candidates with the lowest absolute difference of training and development AUCs.

Two hundred epochs were usually enough for a good convergence. In our experiments, the best model snapshot was generated at the end of epoch 131.

### 2.7 Inference for the deep learning models

Representing nodules in multiple views not only increases the training data size, but also effectively reduces empirical variance of inference by the ensemble trick. In practice, given an input, we forwarded the well‐trained neural network using the mentioned conventional data augmentation (*m* = 3) with 100 runs. Then, probability scores of *EGFR* Mut were obtained by averaging the multiple runs:

$$y_{\text{pred}}=\frac{1}{100}\sum_{i=1}^{100}y_{\text{pred}}^{\left(i\right)}$$

We used the last‐layer (the *DLR* layer in Table 1) 114‐d outputs of the trained 3D DenseNet as the learned representations given nodules, that is, the deep learned radiomics (DLR). More robust DLR was obtained by averaging the feature outputs of multiple runs. Note the DLR and the probability scores could be obtained within a single neural network forward pass.

## 3 RESULTS

### 3.1 Performance of deep learning on *EGFR* mutation status prediction

**Figure 2:**
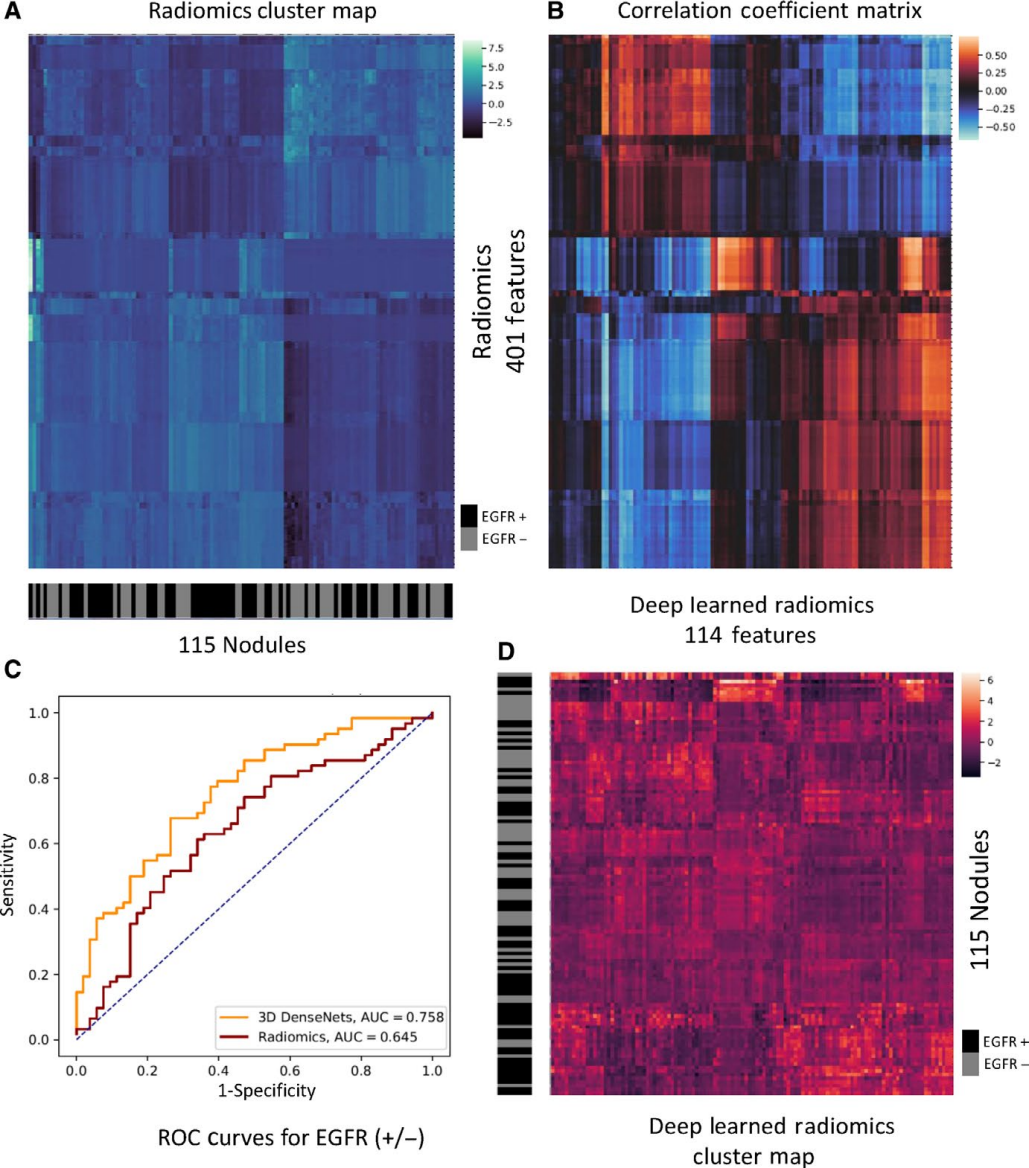
Visualization of conventional radiomics, deep learned radiomics (DLR) and their associations. A, Cluster map of conventional radiomics, with 115 nodules on the *x*‐axis and 401 radiomic features on the *y*‐axis. Each feature was normalized into zero mean and unit standard variance. The nodules of a same cluster (adjacent columns) shared similar radiomic features in Euclidean space. The semantic label *EGFR* Mut/WT of each nodule was shown on the black‐grey bar below the *x*‐axis. B, Correlation coefficient matrix for conventional radiomics and DLR. Note the radiomic features (*y*‐axis in A) and the DLR features (*x*‐axis in D) were both aligned with the correlation coefficient matrix. C, The classification ROC curves of our radiomics‐based and DLR‐based methods. The brighter (red or blue) blocks show the higher correlation. The black denotes no correlation. D, Cluster map of DRL with 115 nodules on the *y*‐axis and 114 radiomic features on the *x*‐axis. Each feature was normalized into zero mean and unit standard variance. The nodules of a same cluster (adjacent rows) shared similar DLR in Euclidean space. The semantic label *EGFR* Mut/WT of each nodule was shown on the black‐grey bar on the left of the *y*‐axis

We compared the performance between deep learning and conventional radiomics on predicting *EGFR* mutation status of lung adenocarcinoma, which implied the associations of *EGFR* genotype and radiomic phenotype. The AUC, which is insensitive of class skews, was used for evaluation. As depicted in Table 3 and Figure 2C, the 3D DenseNets (with *mixup*, ensemble) outperformed our conventional radiomics‐based method (*P* = 0.021, DeLong test^47^). Several prior studies were also listed in Table 3. Since they used nonshared datasets independently, their results were only for reference only. On our holdout test set (HdH test Dataset) containing 115 patients and the TCIA Dataset of 37 patients, 3D DenseNets achieved AUCs of 75.8% and 75.0%, respectively. Considering the class‐imbalance and threshold bias, we believed the threshold‐free AUROC was the most appropriate metric. For reference, we applied a threshold that maximizes the accuracy to obtain the sensitivity, specificity, accuracy, and precision (or positive predictive value). These results were depicted in Table S2. Notably, the data distribution of the TCIA Dataset differed much from that of the HdH Dataset in terms of patients and imaging; the comparable performances on the two datasets indicated the robustness of our method. More importantly, unlike conventional radiomics methods that require labor‐intensive manual segmentation masks, our deep learning method was compatible with approximate locations of the nodules instead.

**Table 3:** Presentation of our method and several previous studies in terms of methods, datasets, and resulting classification AUCs

Method	Training #Patients	Test #Patients	#*EGFR* mut^a^	AUC (%)
Radiomics^23^	353	352	183 (24.0%)^b^	69
+ clinical information				75
Radiomics^17^	298	NA^c^	137 (46.0%)	64.7
+ clinical information				70.9
Radiomics^18^	47	NA^c^	19 (40.4%)	67.0
Radiomics (This study)	464 (HdH train‐dev Dataset)	115 (HdH test Dataset)	62 (53.9%)	64.5
3D DenseNets w/*mixup*, ensemble (This study)	348 (HdH training Dataset)	115 (HdH test Dataset)	62 (53.9%)	75.8
Radiomics (this study)	464 (HdH train‐dev Dataset)	37 (TCIA Dataset)	9 (24.3%)	68.7
3D DenseNets w/*mixup*, ensemble (this study)	348 (HdH training Dataset)	37 (TCIA Dataset)	9 (24.3%)	75.0

a Shown as the number of cases (percentage).

b Estimated using the proportion of *EGFR* Mut on the entire data set, rather than the test set.

c The evaluation results are based on multivariate statistical analysis, rather than the practice of *training – validation (development) – test* in machine learning. Since *the prior studies* listed in the above table used nonshared datasets independently, the results are *for reference only*.

### 3.2 Potential association between the conventional radiomics and deep learned radiomics

Radiomics analysis provides important medical insights. However, conventional radiomics requires manual segmentation, which is a tedious process in practice. Instead, our method based on 3D deep learning predicts the labels automatically with even better performance. To our knowledge, few prior studies compare these two methods together. Assumed that deep learning networks particularly learn the representation of radiomics with end‐to‐end training, we investigated the relation between the both, and discuss the reason why deep learning provided better discriminative performance on this problem.

To this end, we analyzed 401 conventional radiomic features (ICC > 0.8), against 114 deep learned radiomics (DLR) features extracted from 3D DenseNets (see the “Inference for the deep learning models” section). As illustrated in Figure 2A,D, the unsupervised clusters of both the conventional radiomics and DLR matched much with the semantic labels (Mut/WT); in other words, the continuous regions on the black‐grey bars shared numbers of similar features respectively. However, the cluster map of DLR had obviously shaper contrast than that of conventional radiomics, which indicated the DLR features are more representative and compact than the hand‐craft features. It well explained the higher classification performance of DLR than conventional radiomics.

Moreover, we associated DLR and conventional radiomics, by visualizing the pairwise Pearson correlation coefficients on the HdH test Dataset with a cluster map in Figure 2B. Though the radiomic features and DLR features do not highly correlate (correlation coefficients range from −0.5 to +0.5), the cluster map indicated relatively high correlation (bright red or blue) between DLR and radiomics. It is worth noting that, most of the radiomic features were potentially correlated with at least one DLR feature, while certain DLR features showed low correlation with all the radiomic features, which potentially corresponded to the extra high‐level information with more hints on modeling the *EGFR* status. Considering that our DLR‐based method showed better performance, it is reasonable to assume that the DLR features encoded automatically not only almost all information of the (highly correlated) conventional radiomics with even more compact representation (more informative with lower feature dimension), but also critical discriminative information not in the conventional radiomics.

To verify our assumption further, we used the 114 DLR features together with the 401 radiomics features to fit a cross validation searched logistic regression model as in our conventional radiomics analysis. An automatic machine learning tool (AutoML)^36^ was also applied for searching a best‐performing model. The resulting model achieved AUCs of 71.2% and 74.2% on the HdH test Dataset and TCIA Dataset, respectively, which performs *poorer* than using DLR alone. It implied that, combining the conventional radiomics and DLR naïvely could *not* boost the performance, due to the high correlation between the both. Note that the feature selection procedure to reduce the redundancy between the features had been included in the AutoML search procedure.

### 3.3 Ensemble vs vanilla (no ensemble)

The inference‐stage data augmentation by representing a nodule in multiple views, was not only reasonable from a medical perspective, but also made sense in our empirical results. As depicted in Table 4, this simple ensemble technique was beneficial to reducing the inference variance. The vanilla term referred to inference with a single forward. The ensemble inference produced a more stable performance, on the HdH Dataset (training, development, test) and TCIA Dataset, though the vanilla inference might produce even better results in some cases.

**Table 4:** Dataset summary and prediction performance of deep learning systems on HdH Dataset (training, development and test) and TCIA Dataset

Dataset	#Patients	EGFR^+^ a	AUC (w/*mixup*, ensemble) (%)	AUC (w/*mixup*, vanilla) (%)	AUC (w/o *mixup*, ensemble) (%)	AUC (w/o *mixup*, vanilla) (%)
HdH training dataset	348	185 (53.2%)	76.7	76.0	71.0	70.1
HdH development dataset	116	61 (52.6%)	74.1	74.6	69.2	70.4
HdH test dataset	115	62 (53.9%)	75.8	76.8	67.9	67.9
TCIA dataset	37	9 (24.3%)	75.0	68.3	70.6	71.4

a Shown as the number of cases (percentage).

### 3.4 *Mixup* vs no *mixup*

The *mixup* technique was critical for training our 3D DenseNets. As shown in Table 4, the models with *mixup* training significantly outperformed those without in all of our experiments. The models without *mixup* training follows the same model selection procedure.

**Figure 3:**
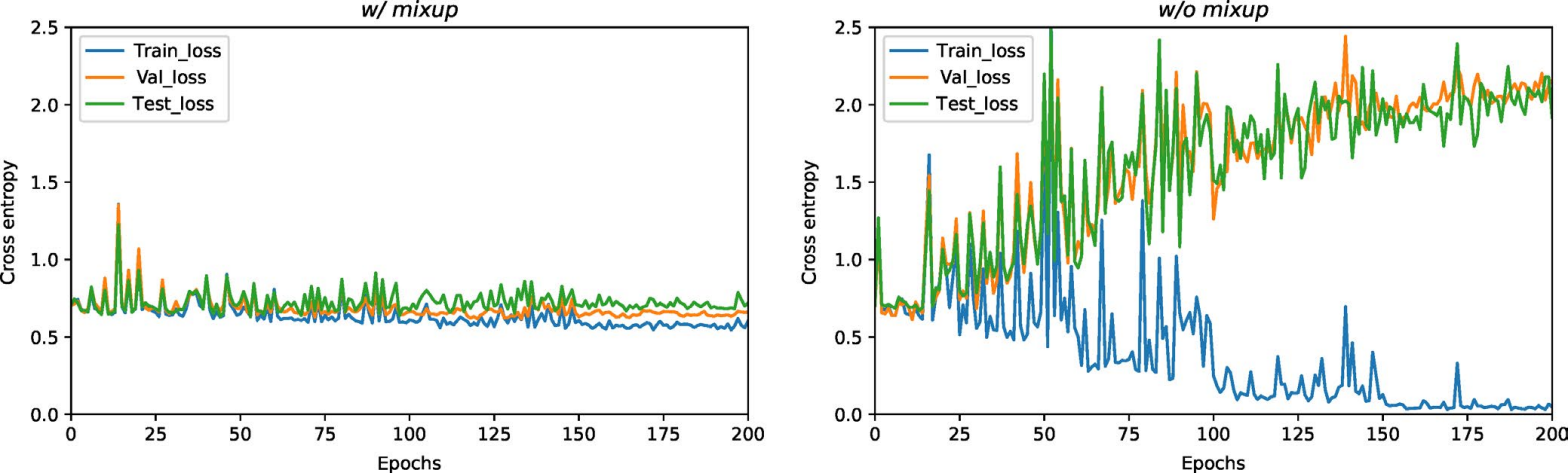
The learning curves of the best models with *mixup* training and those without, in terms of binary cross‐entropy loss. The losses on the HdH training, development (val) set and test Dataset were shown on the figures. “epochs” on the *x*‐axis means the training consumes once the entire training set

We explained the reason for these remarkable differences with the surprisingly strong regularization effects of *mixup* training. As demonstrated in Figure 3, the learning curves with *mixup* training were much smoother, and no severe overfitting was observed. On the contrary, the training without *mixup* was not stable; besides, there existed typical overfitting, making the model selection impractical. Considering the unreasonable effectiveness of *mixup* training, it could potentially be a standard technique in medical image computing in the future.

### 3.5 The t‐SNE visualization of the deep learned radiomics

**Figure 4:**
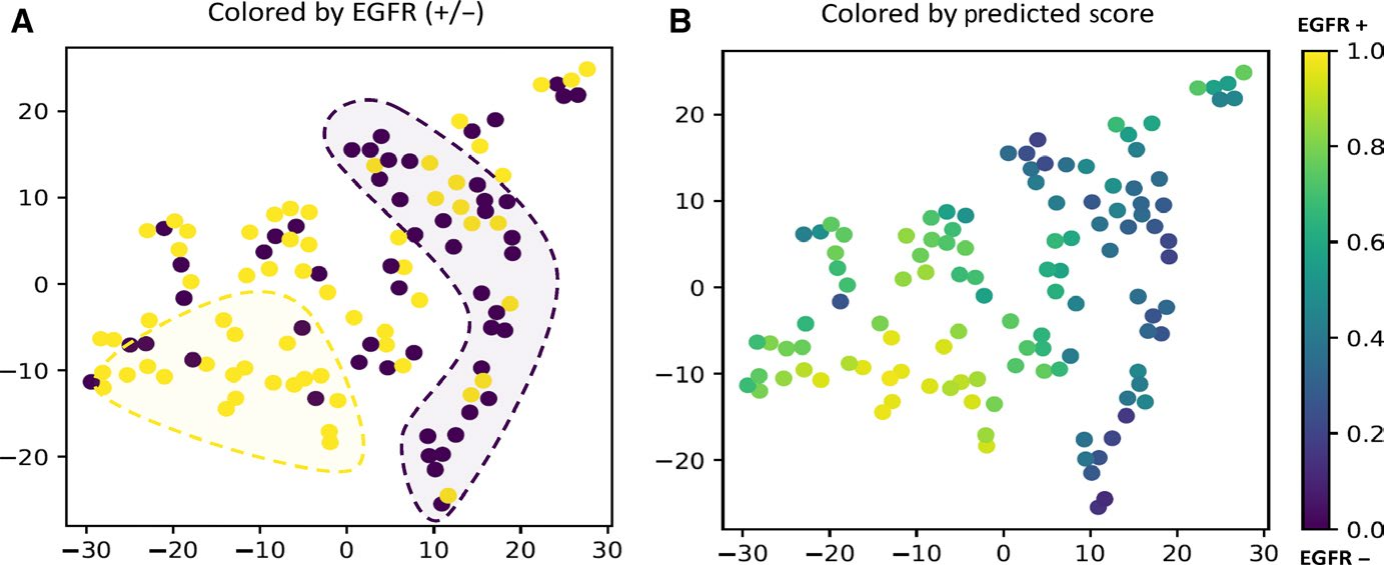
t‐SNE visualization of the deep learned radiomics in a 2D space on the HdH test Dataset. A, The t‐SNE visualization scatter plot colored by the *EGFR* labels. Clusters can be found labeled by the ground truth. B, The t‐SNE visualization scatter plot colored by the *EGFR* probability score predicted by the 3D DenseNets. The prediction scores are consistent with ground truth

To explore the manifold structure of the DLR intuitively, we visualized the DLR features on the HdH test Dataset using t‐Distributed Stochastic Neighbor Embedding (t‐SNE)^48^ as illustrated in Figure 4. No distinct pattern was shown on the t‐SNE visualization, which was reasonable considering the difficulty of using the phenotype information to predict the genotype expression. Even so, two clusters of *EGFR* Mut and *EGFR* WT could be found, see Figure 4. Most of the DLR features outside of the contours of the two clusters were assigned a score with high uncertainty by the 3D DenseNets. Nevertheless, the DLR features were shown to be meaningful and representative despite the difficulty of the task.

## 4 DISCUSSION

This study developed a deep supervised learning approach to predict *EGFR*‐mutant lung adenocarcinoma in CT images. Compared with conventional radiomics, the models are less prone to overfitting on the limited training data and show better prediction performance. The deep learning method is also labor‐saving since it does not require precise segmentation of nodules. More importantly, we empirically find that the deep learning models learn more representative features than conventional radiomics, named *deep learned radiomics (DLR)*, which are the keys to better analytic performances.

Our method outperformed previous studies and established a new methodology on this task. Using imaging information only, our deep 3D DenseNets achieved AUCs of 75.8% and 75.0% on the HdH test Dataset and independent public TCIA Dataset, respectively, which were better than the best of previous radiomics method (69%), and on par with their model combining additional clinical information (75%). We analyzed the associations between DLR and conventional radiomics, and empirically showed that DLR features were more representative and learned extra imaging information compared to conventional radiomics. Furthermore, we introduced the *mixup* training to our deep learning method, which suggested “unreasonable effectiveness” in regularizing the training of the deep neural networks with limited data.

Decoding image phenotypes using radiomics to predict tumor genotypes, called *radio‐genomics* as well, shows promising performances and outcomes in precision medicine research. A few recent studies have investigated radiomics analysis to noninvasively predict *EGFR* mutation status.^16^, ^23^ For example, Rios et al^23^ discriminate *EGFR* Mut and WT cases with an AUC of 69%, with an improvement on AUC (75%) when combining clinical information. Despite these impressive results, radiomics‐based methods are limited with expressiveness of the hand‐craft features; besides, the requirement of manual segmentation makes it difficult to apply in practical clinical contexts.

In contrast, our deep learning method is automatic in predicting the *EGFR* mutation status, which only requires an approximate location of nodule instead of labor‐intensive voxel‐wise segmentation. More importantly, the resulting DLR features are learned automatically with end‐to‐end training. In our association analysis, we found that some DLR features were not correlated with any radiomic features in our experiments, implying extra information extracted by our 3D DenseNets. Powered by dense connections^31^ and *mixup* training,^32^ our method achieved a *state‐of‐the‐art* performance. Considering that sensitizing *EGFR* mutations are more likely found in Asian patients,^2^ a selected subset from TCIA database^33^ was used for testing the generality of our system. Our deep learning system showed robust performance on the public data set.

Radiomic features, defined by image analysis algorithms on numbers of visual characteristics, such as shape, volume, and intensity, are low‐level features without high‐level semantic information. However, these features are naturally “grey‐box” interpretable. On the other hand, DLR features are semantically high‐level thanks to the end‐to‐end learning, yet deep learning models are known to be black‐box artificial intelligence lack of the desirable interpretability, especially in the medical contexts. Our association analysis not only provides insight on understanding the DLR features, but also approaches opening the black‐box of deep learning in medical image computing by a grey‐box model. However, it is hardly possible to identify the specific correlation between radiomics and DLR, we leave this for further exploration.

There were limitations in our study. Due to resource limitation, *EGFR* mutations were detected with ARMS‐PCR covering specific loci, thus *EGFR* mutations were narrowly defined in this study. Further studies with mutations detected by second‐generation sequencing are needed. Besides, information within the CT images is relative limited; integrating more available information of patients, such as clinicopathological facets, blood testing result, proteomics, and even the lifestyles, into the models can be beneficial to inferring the genotypes in multiple views. Recently, liquid biopsy, using blood as opposed to tumor samples for molecular analysis, was developed to identify EGFR mutations with promising results.^49^, ^50^ Taking advantage of these valuable information or integrating different promising approaches may improve the performance of predicting the EGFR mutation status. We consider it as a future direction. Moreover, the sample size of the independent validation was still small, and our deep leaning approach should be tested in larger cohorts. Also, this was a single‐center study, our deep learning systems desire larger datasets with more diversity. Further improvement can be made to practically help scalable precision medicine; however, the methodology established by this study, could serve as a paradigm for future studies. Lastly, only thin‐slice CT images were included in the current study to mitigate the radiomic feature variabilities. However, whether the deep learning method could perform better with different slice thickness CT images is worth further investigating. Actually, this is one of our ongoing research.

In conclusion, the proposed deep learning system predicts EGFR‐mutant lung adenocarcinoma in CT images automatically and noninvasively with promising performance, indicating the potential to help clinical decision‐making by identifying eligible patients of pulmonary adenocarcinoma for EGFR‐targeted therapy. The association study between conventional radiomics and deep learned radiomics discusses the relation between the both, and approaches toward a grey‐box explanation methodology of black‐box model.

## CONFLICT OF INTEREST

The authors declare that they have no conflict of interest.

## AUTHORS CONTRIBUTION

Conception and design: W. Zhao, J. Yang, Y. Hua, M. Li. Development of methodology: W. Zhao, J. Yang, D. Bi, B.Ni, M.Xu, Y. Hua, M. Li. Acquisition of data (provided animals, acquired and managed patients, provided facilities, etc): W. Zhao, J. Yang, X. Zhu, Y. Sun, L. Jin, C. li, P. Gao, Y.Hua, M. Li. Analysis and interpretation of data (eg, statistical analysis, biostatistics, computational analysis):W. Zhao, J. Yang, D.Bi, Y. Hua, M. Li. Writing, review, and/or revision of the manuscript: All authors Administrative, technical, or material support (ie, reporting or organizing data, constructing databases): W. Zhao J. Yang, P. Wang, Y. Hua, M. Li. Study supervision: W. Zhao, B. Ni, P. Wang, Y. Hua, M. Li. Other (algorithm and software development): J. Yang.

## ETHICAL APPROVAL

This retrospective study was approved by the Institutional Review Board of our institution (NO.20170103), which waived the requirement for patients’ informed consent referring to the CIOMS guideline.
