# Supplementary material for: Toward automatic prediction of EGFR mutation status in pulmonary adenocarcinoma with 3D deep learning
Source: Cancer Med. 2019 May 10;8(7):3532–43. doi: 10.1002/cam4.2233 (PMC6601587; doi:10.1002/cam4.2233)
Supplement: Supplementary file 2 [file CAM4-8-3532-s002.docx]

**S2 Table** Sensitivity, specificity, accuracy and positive predictive values of different deep learning systems on HdH Dataset (training, development and test) and TCIA Dataset

| Dataset | *Mixup*, ensemble) | *Mixup*, vanilla | No *mixup*, ensemble | *No mixup*,  vanilla |
| --- | --- | --- | --- | --- |
| Sensitivity |  |  |  |  |
| HdH training Dataset | 61.1% | 73.5% | 74.6% | 64.3% |
| HdH development Dataset | 78.7% | 78.7% | 55.7% | 54.1% |
| HdH test Dataset | 85.5% | 90.3% | 67.7% | 71.0% |
| TCIA Dataset | 88.9% | 44.4% | 55.6% | 44.4% |
| Specificity |  |  |  |  |
| HdH training Dataset | 83.4% | 66.9% | 58.3% | 69.3% |
| HdH development Dataset | 63.6% | 61.8% | 74.6% | 80.0% |
| HdH test Dataset | 52.8% | 49.1% | 66.0% | 58.5% |
| TCIA Dataset | 75.0% | 85.7% | 89.3% | 89.3% |
| Accuracy |  |  |  |  |
| HdH training Dataset | 71.6% | 70.4% | 67.0% | 66.7% |
| HdH development Dataset | 71.6% | 70.7% | 64.7% | 66.4% |
| HdH test Dataset | 70.4% | 71.3% | 67.0% | 65.2% |
| TCIA Dataset | 78.4% | 75.7% | 81.1% | 78.4% |
| Positive predictive values |  |  |  |  |
| HdH training Dataset | 80.7% | 71.6% | 67.0% | 70.4% |
| HdH development Dataset | 70.6% | 69.6% | 70.8% | 75.0% |
| HdH test Dataset | 68.0% | 67.5% | 70.0% | 66.7% |
| TCIA Dataset | 53.3% | 50.0% | 62.5% | 57.1% |
