# Supplementary material for: Toward automatic prediction of EGFR mutation status in pulmonary adenocarcinoma with 3D deep learning
Source: Cancer Med. 2019 May 10;8(7):3532–43. doi: 10.1002/cam4.2233 (PMC6601587; doi:10.1002/cam4.2233)
Supplement: Supplementary file 3 [file CAM4-8-3532-s003.docx]

**Supplementary data**

**CT acquisition parameters**

Preoperative chest CT scans were performed by using the following six scanners: GE Discovery CT750 HD, 64-slice LightSpeed VCT, Revolution CT (GE Medical Systems); Somatom Definition flash, Somatom Sensation-16, Somatom Force (Siemens Medical Solutions) with the following parameters: 120 kVp; 100– 200 mAs; pitch, 0.75–1.5; and collimation, 0.75–1.5 mm respectively. All imaging data were reconstructed using a medium sharp reconstruction algorithm with a thickness of 0.75–1.5 mm. In all patients, CT images were acquired in the supine position at full inspiration.

**Radiomics extraction methodology**

**(1) Normalization processing**

Because of the differences in pixel spacing and slice thickness, the images and tumor contours were subsequently normalized to isometric voxels (0.25mm). This step based on the linear interpolation was calculated by the Matlab 2016b.

**(2) Image filtration**

A Laplacian of Gaussian spatial band-pass filter (∇2*G*) was used to derive image features at different spatial scales by turning the filter parameter from 1.0 to 2.5 (1.0, 1.5, 2.0 and 2.5). The Laplacian of Gaussian filter distribution is given by

$$\nabla^{2}G\left( x,y \right)=\frac{-1}{\pi\sigma^{4}}\left( 1-\frac{x^{2}+y^{2}}{2\sigma^{2}} \right)e^{-(\frac{x^{2}+y^{2}}{2\sigma^{2}})}$$

where σ is the value of the filter parameter and x, y denote the spatial coordinates of the pixel.

**(3) Feature extraction**

A series of gray-level histogram features, gray-level co-occurrence matrix features and gray-level run lengths matrix features, as listed in **Table 1**, were generated from the images obtained without and with filtration.

**Table 1. Extracted imaging features**

| **Gray-level histogram feature** | **Co-occurrence matrix** | **Run lengths matrix** |
| --- | --- | --- |
| skewness_σ | Contrast θ_φ_σ | SRE_α_σ |
| kurtosis_σ | Correlation θ_φ_σ | LRE_α_σ |
| mean_σ | Entropy θ_φ_σ | GLN_α_σ |
| SD_σ | Energy θ_φ_σ | RLN_α_σ |
| mean_ β_σ | Homogeneity θ_φ_σ | RP_α_σ |
| SD_ β _σ |  |  |

σ denotes the filter value, which may be 0, 1.0, 1.5, 2.0 or 2.5.

α, θ, φ denotes the considered direction, which may be 0º, 45º, 90º or 135º.

β denotes the top percentage of the histogram curve, which may be 10%, 25% or 50%.

**1) Gray-level histogram features** describe the gray level distribution of the image.

| **Feature** | **Description** | **Formula** |
| --- | --- | --- |
| mean | Average value of the histogram. | $mean=\frac{1}{N}\sum_{i=1}^{N} X(i)$ |
| SD | Stability of the gray level histogram. | $SD=\frac{1}{N}\sum_{i=1}^{N} {(X\left( i \right)-\bar{X})}^{2}$ |
| mean_ β | Percentile mean calculated from the top 50%, 25%, and 10% of the histogram curve. | $mean \_\beta=\frac{1}{N-M}\sum_{i=M}^{N} X（i）$ |
| SD_ β | Percentile SD calculated from the top 50%, 25%, and 10% of the histogram curve. | $SD \_\beta=\frac{1}{N-M}\sum_{i=M}^{N} (X\left（ i \right）-{\bar{X})}^{2}$ |
| kurtosis | Sharpness of the histogram. | $kurtosis=\frac{\frac{1}{N}\sum_{i=1}^{N} (X\left( i \right)-{\bar{X})}^{4}}{({\sqrt{\frac{1}{N}\sum_{i=1}^{N} (X\left( i \right)-{\bar{X})}^{2}})}^{4}}$ |
| skewness | Degree of asymmetry around the mean value. | $skewness\mathbf{=}\frac{\frac{1}{N}\sum_{i=1}^{N} (X\left( i \right)-{\bar{X})}^{3}}{({\sqrt{\frac{1}{N}\sum_{i=1}^{N} (X\left( i \right)-{\bar{X})}^{2}})}^{3}}$ |

**2) The Gray-level Co-occurrence Matrix (GLCM)** is a second-order statistical texture feature that is defined as matrix Pδ(i,j) to indicate the relative frequency of the intensity values of two pixels (i and j) with distance1 and 13 different directions. θ is the angle interval between a direction and the positive X direction in XY plane, and φ is the angle interval between a direction and the positive Z direction.

| **Direction (θ，φ)** | **Displacement vector** | **Corresponding duplicate vector** |
| --- | --- | --- |
| (0°，45°) | （1,0,1） | (-1,0,-1) |
| (0°，90°) | （1,0,0） | (-1,0,0) |
| (0°，135°) | （1,0,-1） | (-1,0, 1) |
| (45°，45°) | （1,1,1） | (-1,-1,-1) |
| (45°，90°) | （1,1,0） | (-1,-1,0) |
| (45°，135°) | （1,1,-1） | (-1,-1,1) |
| (90°，45°) | （0,1,1） | (0,-1,-1) |
| (90°，90°) | （0,1,0） | (0,-1,0) |
| (90°，135°) | （0,1,-1） | (0,-1,1) |
| (135°，45°) | （-1,1,1） | (1,-1,-1) |
| (135°，90°) | （-1,1,0） | (1,-1,0) |
| (135°，135°) | （-1,1,-1） | (1,-1,1) |
| (-,0°) | （0,0,1） | (0,0,-1) |

P (𝑖, 𝑗) is the co-occurrence matrix, Ng is the number of discrete intensity levels in the image, 𝜇 is the mean of P (𝑖, 𝑗), 𝜇x (𝑖) is the mean of 𝑃𝑥 (𝑖), 𝜇y (𝑗) is the mean of 𝑃𝑦 (𝑗), σ(𝑖) is the standard deviation of (𝑖), and σ(𝑗) is the standard deviation of (𝑗).

| **Feature** | **Description** | **Formula** |
| --- | --- | --- |
| contrast | Uniformity of the image grayscale distribution and degree of thickness in texture. | $contrast=\sum_{i=1}^{Ng} \sum_{j=1}^{Ng} \left\vert i-j \right\vert^{2}P(i,j)$ |
| correlation | Measurement of the gray-level linear dependence between pixels at specified positions relative to each other. | $correlation=\frac{\sum_{i=1}^{Ng} \sum_{j=1}^{Ng} ijP\left( i,j \right)-\mu_{i}(i)\mu_{i}(j)}{\sigma_{x}\left( i \right)\sigma_{y}(j)}$ |
| entropy | Homogeneity of an image. Inhomogeneous images have low entropy, whereas a homogeneous scene has high entropy. | $entropy=-\sum_{i=1}^{Ng} \sum_{j=1}^{Ng} P\left( i,j \right)log[P(i,j)]$ |
| energy | Sum of squares of entries in the GLCM. Measurement of the image homogeneity. | $energy=\sum_{i=1}^{Ng} \sum_{j=1}^{Ng} {[P(i,j)]}^{2}$ |
| homogeneity | Local homogeneity. | $homogeneity=\sum_{i=1}^{Ng} \sum_{j=1}^{Ng} \frac{P\left( i,j \right)}{1+\left\vert i-j \right\vert^{2}}$ |

**3) The Gray-level Run Lengths Matrix (GLRLM)** is a second-order statistical texture feature. It is defined as matrix Pδ (i, j) to indicate the number of times that the image contains a run of length j, in four directions (0º, 45º, 90º, and 135º), consisting of points having gray level i.

P (i, j) is the (i, j)th entry in the given run length matrix; N0 is the number of gray level in the picture; Nr is the number of different run lengths that occur(so that the matrix is N0 by Nr); P is the number of points in the picture.

| **Feature** | **Description** | **Formula** |
| --- | --- | --- |
| Short Runs Emphasis  (SRE) | Divides each run length value by the length of the run squared | $SRE=\sum_{i=1}^{N_{0}} \sum_{j=1}^{N_{r}} \frac{P(i,j)}{j^{2}}/\sum_{i=1}^{N_{0}} \sum_{j=1}^{N_{r}} P(i,j)$ |
| Long Runs Emphasis  (LRE) | Multiplies each run length value by the length of the run squared | $LRE=\sum_{i=1}^{N_{0}} \sum_{j=1}^{N_{r}} j^{2}P(i,j)/\sum_{i=1}^{N_{0}} \sum_{j=1}^{N_{r}} P(i,j)$ |
| Gray Level Nonuniformity  (GLN) | Squares the number of run lengths for each gray level. The sum of the squares is divided by the normalizing factor of the total number of runs. | $GLN=\sum_{i=1}^{N_{0}} [\sum_{j=1}^{N_{r}} P\left( i,j \right)]^{2}/\sum_{i=1}^{N_{0}} \sum_{j=1}^{N_{r}} P(i,j)$ |
| Run Length Nonuniformity  (RLN) | Squares the number of run lengths for each length. The sum of the squares is divided by the normalizing factor | $RLN=\sum_{j=1}^{N_{r}} [\sum_{i=1}^{N_{0}} P\left( i,j \right)]^{2}/\sum_{i=1}^{N_{0}} \sum_{j=1}^{N_{r}} P(i,j)$ |
| Run Percentage  (RP) | A ratio of the total number of runs to the total number of possible runs if all runs had a length of one | $RP=\sum_{i=1}^{N_{0}} \sum_{j=1}^{N_{r}} P(i,j)/P$ |
